# *catena*-Poly[[(2,2′-bipyridine)­manganese(II)]-μ_3_-4,4′-sulfonyl­dibenzoato]

**DOI:** 10.1107/S160053681101631X

**Published:** 2011-05-07

**Authors:** Shi-Wei Yan, Guang-Ju Zhang, Hai-Yan Chen, Suo-Cheng Chang, Fu-Tian Zhang

**Affiliations:** a College of Chemistry and Chemical Engineering, Southwest University, Chongqing 400715, People’s Republic of China

## Abstract

In the title compound, [Mn(C_14_H_8_O_6_S)(C_10_H_8_N_2_)]_*n*_, the Mn^II^ ion is coordinated by four O atoms from three 4,4′-sulfonyl­dibenzoate (sdba) ligands and two N atoms from one 2,2′-bipyridine (2,2′-bipy) ligand in a distorted octa­hedral geometry. The manganese atoms are alternately bridged either by two sdba ligands, with an Mn⋯Mn separation of 12.284 (1) Å, or by two carboxyl­ate groups from two sdba ligands, with an Mn⋯Mn separation of 4.064 (1) Å, thus producing polymeric chains propagated in [101]. Weak inter­molecular C—H⋯O hydrogen bonds and π–π inter­actions [centroid–centroid distance of 3.730 (3) Å between the aromatic rings of neighbouring polymeric chains] further stabilize the crystal packing.

## Related literature

For the crystal structures of related Mn^II^ complexes with sdba ligands, see: Li *et al.* (2010 ▶); Xiao *et al.* (2008 ▶).
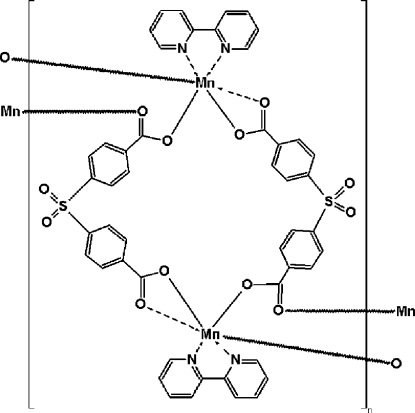


## Experimental

### 

#### Crystal data

[Mn(C_14_H_8_O_6_S)(C_10_H_8_N_2_)]
*M* *_r_* = 515.39
Monoclinic, 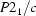

*a* = 12.302 (3) Å
*b* = 15.386 (3) Å
*c* = 12.255 (3) Å
β = 111.06 (3)°
*V* = 2164.7 (10) Å^3^
*Z* = 4
Mo *K*α radiation
μ = 0.75 mm^−1^
*T* = 293 K
0.52 × 0.47 × 0.23 mm

#### Data collection

Rigaku R-AXIS RAPID IP diffractometer
Absorption correction: multi-scan (*ABSCOR*; Higashi, 1995 ▶) *T*_min_ = 0.696, *T*_max_ = 0.846
17821 measured reflections
4193 independent reflections
3209 reflections with *I* > 2σ(*I*)
*R*_int_ = 0.071

#### Refinement

*R*[*F* ^2^ > 2σ(*F* ^2^)] = 0.048
*wR*(*F* ^2^) = 0.130
*S* = 1.06
4193 reflections
307 parameters
H-atom parameters constrained
Δρ_max_ = 0.36 e Å^−3^
Δρ_min_ = −0.45 e Å^−3^

### 

Data collection: *PROCESS-AUTO* (Rigaku, 1998 ▶); cell refinement: *PROCESS-AUTO*; data reduction: *CrystalStructure* (Rigaku/MSC, 2002 ▶); program(s) used to solve structure: *SHELXS97* (Sheldrick, 2008 ▶); program(s) used to refine structure: *SHELXL97* (Sheldrick, 2008 ▶); molecular graphics: *SHELXTL-Plus* (Sheldrick, 2008 ▶); software used to prepare material for publication: *SHELXL97*.

## Supplementary Material

Crystal structure: contains datablocks I, global. DOI: 10.1107/S160053681101631X/cv5080sup1.cif

Structure factors: contains datablocks I. DOI: 10.1107/S160053681101631X/cv5080Isup2.hkl

Additional supplementary materials:  crystallographic information; 3D view; checkCIF report

## Figures and Tables

**Table 1:** Hydrogen-bond geometry (Å, °)

*D*—H⋯*A*	*D*—H	H⋯*A*	*D*⋯*A*	*D*—H⋯*A*
C1—H1*A*⋯O5^i^	0.93	2.44	3.123 (4)	131
C16—H16*A*⋯O6^ii^	0.93	2.59	3.424 (4)	149
C19—H19*A*⋯O2^iii^	0.93	2.54	3.210 (4)	129
C21—H21*A*⋯O5^iv^	0.93	2.51	3.337 (4)	148

Symmetry codes: (i) 
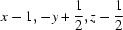
; (ii) 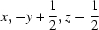; (iii) 
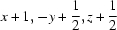
; (iv) 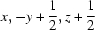.

**Fig. 1.**
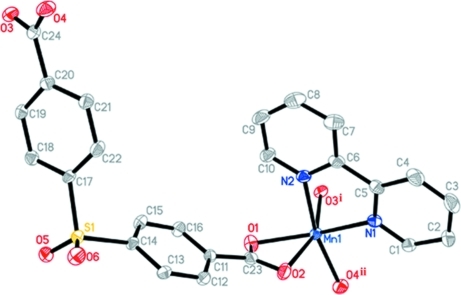
The coordination environment of MnII in (I) showing the atomic numbering and 30% probability displacement ellipsoids [symmetry codes: (i) -x + 2, -y, -z + 1; (ii) x - 1, y, z - 1]. H atoms omitted for clarity.

**Fig. 2.**
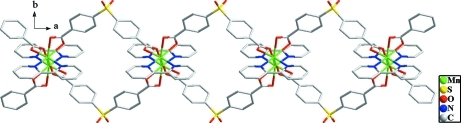
A portion of the polymeric chain in (I) viewed along c axis.

## supplementary crystallographic information

### Comment

4,4'-Sulfonyldibenzoic acid (H_2_sdba) is a typical V-shaped dicarboxylate ligand, which is important in construction of some novel frameworks with Mn salts (Li *et al.*, 2010; Xiao *et al.*, 2008). Here we report the crystal structure of the title compound, [Mn(sdba)(2,2'-bipy)]_n_ (I).

As shown in Figure 1, the crystallographically independent Mn^II^ atom exhibits a distorted octahedral geometry, being coordinated with two nitrogen atoms from one 2,2'-bipy ligand (Mn1—N1=2.230 (2) Å, Mn1—N1=2.315 (3) Å) and four oxygen atoms of three sdba ligands (Mn1—O1=2.159 (2) Å, Mn1—O2=2.445 (2) Å, Mn1—O3^í^=2.119 (2) Å, Mn—O4^íí^=2.125 (3) Å). The sdba ligand acts as a tetradentate ligand, as one carboxylate group adopts a bidentate bridging mode connecting two Mn^II^ ions, while the other carboxylate group adopts a bidentate chelating coordination mode connecting one Mn^II^ ion. Two Mn^II^ centers are bridged by two carboxylate groups of different sdba ligands to yield a dinuclear manganese core with Mn···Mn of 4.064 (1) Å. Then the dinuclear manganese units are extended by sdba ligands to generate a one-dimensional double-chain along *c* axis (Figure 2), and the 2,2'-bipy ligands are chelated on both sides of the double-chain. The one-dimensional double-chains are further linked by weak π—π stacking with the distance of 3.730 (3)Å between the centroids of aromatic rings from the neighbouring polymeric chains and intermolecular C—H···O hydrogen-bonding interactions (Table 1) to form a three-dimensional supramolecular network.

### Experimental

A mixture of Mn(CH_3_COO)_2_.4H_2_O (0.184 g, 0.75 mmol), H_2_sdba (0.153 g, 0.50 mmol), 2,2'-bipy (0.078 g, 0.5 mmol) and water (10 ml) was stirred about 15 min in air, then transferred and sealed in an 18 ml Teflon-lined autoclave, which was heated at 160 °C for 60 h. After slow cooling to the room temperature, paleyellow block crystals of I were filtered off, washed with distilled water, and dried at ambient temperature.

### Refinement

C-bound H atoms were positioned geometrically and refined as riding, with C—H = 0.93 Å, and *U*_iso_(H) = 1.2*U*_eq_(C).

### Crystal data

**Table:**

[Mn(C_14_H_8_O_6_S)(C_10_H_8_N_2_)]	*F*(000) = 1052
*M**_r_* = 515.39	*D*_x_ = 1.581 Mg m^−^^3^
Monoclinic, *P*2_1_/*c*	Mo *K*α radiation, λ = 0.71073 Å
Hall symbol: -P 2ybc	Cell parameters from 17821 reflections
*a* = 12.302 (3) Å	θ = 3.2–26.0°
*b* = 15.386 (3) Å	µ = 0.75 mm^−^^1^
*c* = 12.255 (3) Å	*T* = 293 K
β = 111.06 (3)°	Block, yellow
*V* = 2164.7 (10) Å^3^	0.52 × 0.47 × 0.23 mm
*Z* = 4	

### Data collection

**Table:**

Rigaku R-AXIS RAPID IP diffractometer	4193 independent reflections
Radiation source: fine-focus sealed tube	3209 reflections with *I* > 2σ(*I*)
graphite	*R*_int_ = 0.071
Detector resolution: 100x100 microns pixels mm^-1^	θ_max_ = 26.0°, θ_min_ = 3.2°
Oscillation scans	*h* = −14→15
Absorption correction: multi-scan (*ABSCOR*; Higashi, 1995)	*k* = −18→18
*T*_min_ = 0.696, *T*_max_ = 0.846	*l* = −14→15
17821 measured reflections	

### Refinement

**Table:**

Refinement on *F*^2^	Primary atom site location: structure-invariant direct methods
Least-squares matrix: full	Secondary atom site location: difference Fourier map
*R*[*F*^2^ > 2σ(*F*^2^)] = 0.048	Hydrogen site location: inferred from neighbouring sites
*wR*(*F*^2^) = 0.130	H-atom parameters constrained
*S* = 1.06	*w* = 1/[σ^2^(*F*_o_^2^) + (0.073*P*)^2^] where *P* = (*F*_o_^2^ + 2*F*_c_^2^)/3
4193 reflections	(Δ/σ)_max_ < 0.001
307 parameters	Δρ_max_ = 0.36 e Å^−^^3^
0 restraints	Δρ_min_ = −0.45 e Å^−^^3^

### Special details

**Table:**

Geometry. All e.s.d.'s (except the e.s.d. in the dihedral angle between two l.s. planes) are estimated using the full covariance matrix. The cell e.s.d.'s are taken into account individually in the estimation of e.s.d.'s in distances, angles and torsion angles; correlations between e.s.d.'s in cell parameters are only used when they are defined by crystal symmetry. An approximate (isotropic) treatment of cell e.s.d.'s is used for estimating e.s.d.'s involving l.s. planes.
Refinement. Refinement of *F*^2^ against ALL reflections. The weighted *R*-factor *wR* and goodness of fit *S* are based on *F*^2^, conventional *R*-factors *R* are based on *F*, with *F* set to zero for negative *F*^2^. The threshold expression of *F*^2^ > σ(*F*^2^) is used only for calculating *R*-factors(gt) *etc*. and is not relevant to the choice of reflections for refinement. *R*-factors based on *F*^2^ are statistically about twice as large as those based on *F*, and *R*- factors based on ALL data will be even larger.

### Fractional atomic coordinates and isotropic or equivalent isotropic displacement parameters (Å^2^)

**Table:**

	*x*	*y*	*z*	*U*_iso_*/*U*_eq_	
Mn1	0.49352 (4)	0.02781 (3)	0.15966 (4)	0.02695 (16)	
S1	1.01353 (7)	0.35180 (5)	0.61927 (7)	0.0286 (2)	
O1	0.6349 (2)	0.11853 (15)	0.18823 (19)	0.0401 (6)	
O2	0.5179 (2)	0.15851 (15)	0.2796 (2)	0.0443 (6)	
O3	1.45788 (19)	0.08393 (14)	0.91298 (19)	0.0345 (5)	
O4	1.3476 (2)	0.07830 (16)	1.0213 (2)	0.0424 (6)	
O5	1.0677 (2)	0.40116 (14)	0.5534 (2)	0.0388 (6)	
O6	0.9747 (2)	0.39560 (15)	0.7020 (2)	0.0411 (6)	
N1	0.3794 (2)	−0.03320 (16)	0.2443 (2)	0.0304 (6)	
N2	0.6113 (2)	−0.03030 (17)	0.3370 (2)	0.0333 (6)	
C1	0.2629 (3)	−0.0305 (2)	0.1955 (3)	0.0359 (8)	
H1A	0.2291	−0.0036	0.1232	0.043*	
C2	0.1911 (3)	−0.0662 (2)	0.2481 (4)	0.0466 (9)	
H2A	0.1105	−0.0629	0.2127	0.056*	
C3	0.2424 (4)	−0.1066 (3)	0.3542 (4)	0.0549 (11)	
H3A	0.1965	−0.1319	0.3915	0.066*	
C4	0.3608 (4)	−0.1097 (2)	0.4049 (3)	0.0510 (10)	
H4A	0.3958	−0.1364	0.4772	0.061*	
C5	0.4288 (3)	−0.0729 (2)	0.3485 (3)	0.0340 (7)	
C6	0.5580 (3)	−0.0731 (2)	0.3980 (3)	0.0361 (8)	
C7	0.6211 (4)	−0.1156 (3)	0.5023 (3)	0.0588 (11)	
H7A	0.5834	−0.1453	0.5445	0.071*	
C8	0.7400 (4)	−0.1125 (3)	0.5405 (4)	0.0697 (13)	
H8A	0.7837	−0.1406	0.6095	0.084*	
C9	0.7959 (4)	−0.0685 (3)	0.4788 (3)	0.0536 (10)	
H9A	0.8768	−0.0656	0.5048	0.064*	
C10	0.7275 (3)	−0.0287 (2)	0.3766 (3)	0.0412 (8)	
H10A	0.7641	0.0008	0.3331	0.049*	
C11	0.7107 (3)	0.21431 (19)	0.3492 (3)	0.0285 (7)	
C12	0.6908 (3)	0.2744 (2)	0.4254 (3)	0.0321 (7)	
H12A	0.6148	0.2872	0.4187	0.039*	
C13	0.7820 (3)	0.3151 (2)	0.5103 (3)	0.0305 (7)	
H13A	0.7683	0.3549	0.5609	0.037*	
C14	0.8948 (3)	0.2955 (2)	0.5190 (2)	0.0262 (6)	
C15	0.9163 (3)	0.2374 (2)	0.4434 (3)	0.0338 (7)	
H15A	0.9924	0.2255	0.4495	0.041*	
C16	0.8244 (3)	0.1971 (2)	0.3591 (3)	0.0336 (7)	
H16A	0.8387	0.1578	0.3081	0.040*	
C17	1.1132 (3)	0.26961 (19)	0.6961 (2)	0.0260 (6)	
C18	1.2271 (3)	0.2711 (2)	0.6983 (3)	0.0305 (7)	
H18A	1.2484	0.3092	0.6506	0.037*	
C19	1.3081 (3)	0.2150 (2)	0.7727 (3)	0.0291 (7)	
H19A	1.3840	0.2146	0.7738	0.035*	
C20	1.2769 (3)	0.15883 (19)	0.8460 (3)	0.0284 (7)	
C21	1.1625 (3)	0.1570 (2)	0.8407 (3)	0.0338 (7)	
H21A	1.1410	0.1189	0.8883	0.041*	
C22	1.0801 (3)	0.2115 (2)	0.7651 (3)	0.0349 (8)	
H22A	1.0030	0.2093	0.7606	0.042*	
C23	0.6138 (3)	0.1627 (2)	0.2652 (3)	0.0332 (7)	
C24	1.3668 (3)	0.10263 (19)	0.9323 (3)	0.0300 (7)	

### Atomic displacement parameters (Å^2^)

**Table:**

	*U*^11^	*U*^22^	*U*^33^	*U*^12^	*U*^13^	*U*^23^
Mn1	0.0240 (3)	0.0330 (3)	0.0205 (3)	0.0005 (2)	0.0039 (2)	0.00292 (18)
S1	0.0243 (4)	0.0299 (4)	0.0269 (4)	−0.0003 (3)	0.0035 (3)	0.0001 (3)
O1	0.0451 (15)	0.0457 (14)	0.0293 (12)	−0.0120 (12)	0.0130 (12)	−0.0043 (10)
O2	0.0273 (13)	0.0462 (15)	0.0557 (16)	−0.0033 (11)	0.0103 (13)	−0.0072 (12)
O3	0.0287 (13)	0.0368 (13)	0.0346 (13)	0.0064 (10)	0.0071 (11)	0.0001 (9)
O4	0.0376 (14)	0.0558 (16)	0.0307 (13)	0.0051 (12)	0.0084 (12)	0.0147 (11)
O5	0.0340 (13)	0.0359 (13)	0.0423 (13)	−0.0044 (10)	0.0085 (12)	0.0096 (10)
O6	0.0385 (14)	0.0440 (14)	0.0378 (13)	0.0024 (11)	0.0101 (12)	−0.0103 (11)
N1	0.0351 (16)	0.0321 (15)	0.0237 (13)	0.0026 (12)	0.0104 (13)	0.0033 (10)
N2	0.0330 (16)	0.0319 (14)	0.0277 (14)	0.0032 (12)	0.0021 (13)	0.0042 (11)
C1	0.034 (2)	0.0343 (18)	0.0365 (19)	0.0025 (15)	0.0096 (17)	0.0032 (14)
C2	0.041 (2)	0.044 (2)	0.061 (2)	−0.0047 (17)	0.026 (2)	0.0050 (18)
C3	0.063 (3)	0.053 (2)	0.061 (3)	−0.010 (2)	0.037 (2)	0.009 (2)
C4	0.063 (3)	0.052 (2)	0.042 (2)	−0.002 (2)	0.023 (2)	0.0146 (18)
C5	0.044 (2)	0.0303 (17)	0.0280 (16)	−0.0021 (15)	0.0130 (16)	0.0014 (13)
C6	0.044 (2)	0.0348 (18)	0.0235 (16)	0.0024 (15)	0.0051 (16)	0.0065 (13)
C7	0.060 (3)	0.068 (3)	0.039 (2)	0.007 (2)	0.006 (2)	0.0247 (19)
C8	0.062 (3)	0.080 (3)	0.046 (3)	0.010 (3)	−0.007 (2)	0.026 (2)
C9	0.039 (2)	0.056 (2)	0.046 (2)	0.0076 (19)	−0.0093 (19)	0.0027 (19)
C10	0.035 (2)	0.043 (2)	0.0373 (19)	0.0025 (16)	0.0027 (18)	−0.0021 (15)
C11	0.0275 (17)	0.0286 (16)	0.0259 (16)	−0.0025 (13)	0.0051 (14)	0.0048 (12)
C12	0.0255 (17)	0.0351 (18)	0.0340 (17)	0.0016 (14)	0.0086 (15)	0.0032 (13)
C13	0.0268 (17)	0.0313 (16)	0.0318 (17)	0.0047 (13)	0.0087 (15)	0.0026 (13)
C14	0.0219 (16)	0.0326 (16)	0.0198 (14)	−0.0002 (13)	0.0022 (13)	0.0029 (12)
C15	0.0238 (17)	0.0431 (19)	0.0352 (18)	0.0014 (15)	0.0115 (15)	−0.0003 (14)
C16	0.0306 (18)	0.0398 (18)	0.0292 (17)	−0.0008 (15)	0.0093 (16)	−0.0057 (13)
C17	0.0220 (16)	0.0310 (16)	0.0208 (15)	0.0010 (13)	0.0025 (13)	0.0013 (12)
C18	0.0305 (18)	0.0329 (17)	0.0291 (17)	−0.0033 (14)	0.0118 (15)	0.0022 (13)
C19	0.0220 (16)	0.0342 (17)	0.0308 (16)	−0.0031 (13)	0.0092 (14)	−0.0008 (13)
C20	0.0298 (17)	0.0326 (17)	0.0203 (15)	0.0005 (13)	0.0059 (14)	0.0007 (12)
C21	0.0296 (18)	0.0421 (19)	0.0273 (17)	−0.0027 (15)	0.0070 (15)	0.0087 (14)
C22	0.0224 (17)	0.049 (2)	0.0330 (18)	−0.0004 (15)	0.0097 (15)	0.0080 (15)
C23	0.0331 (19)	0.0301 (17)	0.0304 (17)	−0.0010 (14)	0.0042 (16)	0.0046 (13)
C24	0.0276 (17)	0.0313 (17)	0.0265 (16)	−0.0021 (14)	0.0043 (14)	−0.0009 (13)

### Geometric parameters (Å, °)

**Table:**

Mn1—O3^i^	2.119 (2)	C6—C7	1.396 (5)
Mn1—O4^ii^	2.125 (3)	C7—C8	1.367 (6)
Mn1—O1	2.159 (2)	C7—H7A	0.9300
Mn1—N1	2.230 (2)	C8—C9	1.370 (6)
Mn1—N2	2.315 (3)	C8—H8A	0.9300
Mn1—O2	2.445 (2)	C9—C10	1.376 (5)
Mn1—C23	2.607 (3)	C9—H9A	0.9300
S1—O6	1.434 (2)	C10—H10A	0.9300
S1—O5	1.435 (2)	C11—C16	1.386 (4)
S1—C14	1.761 (3)	C11—C12	1.397 (4)
S1—C17	1.777 (3)	C11—C23	1.493 (4)
O1—C23	1.263 (4)	C12—C13	1.376 (5)
O2—C23	1.256 (4)	C12—H12A	0.9300
O3—C24	1.259 (4)	C13—C14	1.386 (4)
O3—Mn1^i^	2.119 (2)	C13—H13A	0.9300
O4—C24	1.252 (4)	C14—C15	1.380 (4)
O4—Mn1^iii^	2.125 (3)	C15—C16	1.376 (5)
N1—C1	1.341 (4)	C15—H15A	0.9300
N1—C5	1.347 (4)	C16—H16A	0.9300
N2—C6	1.333 (4)	C17—C22	1.389 (4)
N2—C10	1.335 (4)	C17—C18	1.392 (4)
C1—C2	1.381 (4)	C18—C19	1.385 (4)
C1—H1A	0.9300	C18—H18A	0.9300
C2—C3	1.374 (6)	C19—C20	1.396 (4)
C2—H2A	0.9300	C19—H19A	0.9300
C3—C4	1.363 (6)	C20—C21	1.386 (4)
C3—H3A	0.9300	C20—C24	1.499 (4)
C4—C5	1.383 (5)	C21—C22	1.384 (5)
C4—H4A	0.9300	C21—H21A	0.9300
C5—C6	1.484 (5)	C22—H22A	0.9300
			
O3^i^—Mn1—O4^ii^	104.26 (9)	C8—C7—H7A	120.9
O3^i^—Mn1—O1	105.09 (9)	C6—C7—H7A	120.9
O4^ii^—Mn1—O1	107.06 (10)	C7—C8—C9	121.0 (4)
O3^i^—Mn1—N1	99.99 (9)	C7—C8—H8A	119.5
O4^ii^—Mn1—N1	91.88 (9)	C9—C8—H8A	119.5
O1—Mn1—N1	143.33 (9)	C8—C9—C10	117.3 (4)
O3^i^—Mn1—N2	84.55 (9)	C8—C9—H9A	121.4
O4^ii^—Mn1—N2	162.73 (9)	C10—C9—H9A	121.4
O1—Mn1—N2	84.34 (10)	N2—C10—C9	123.2 (4)
N1—Mn1—N2	71.77 (10)	N2—C10—H10A	118.4
O3^i^—Mn1—O2	158.13 (9)	C9—C10—H10A	118.4
O4^ii^—Mn1—O2	93.73 (9)	C16—C11—C12	118.8 (3)
O1—Mn1—O2	56.85 (8)	C16—C11—C23	119.3 (3)
N1—Mn1—O2	91.62 (8)	C12—C11—C23	121.7 (3)
N2—Mn1—O2	81.55 (9)	C13—C12—C11	121.0 (3)
O3^i^—Mn1—C23	131.24 (10)	C13—C12—H12A	119.5
O4^ii^—Mn1—C23	105.72 (10)	C11—C12—H12A	119.5
O1—Mn1—C23	28.83 (9)	C12—C13—C14	118.8 (3)
N1—Mn1—C23	116.38 (10)	C12—C13—H13A	120.6
N2—Mn1—C23	77.85 (10)	C14—C13—H13A	120.6
O2—Mn1—C23	28.56 (9)	C15—C14—C13	121.1 (3)
O6—S1—O5	119.26 (14)	C15—C14—S1	118.4 (2)
O6—S1—C14	108.70 (14)	C13—C14—S1	120.2 (2)
O5—S1—C14	107.69 (14)	C16—C15—C14	119.5 (3)
O6—S1—C17	107.56 (14)	C16—C15—H15A	120.2
O5—S1—C17	107.65 (14)	C14—C15—H15A	120.2
C14—S1—C17	105.11 (15)	C15—C16—C11	120.7 (3)
C23—O1—Mn1	95.67 (19)	C15—C16—H16A	119.6
C23—O2—Mn1	82.89 (19)	C11—C16—H16A	119.6
C24—O3—Mn1^i^	132.0 (2)	C22—C17—C18	120.8 (3)
C24—O4—Mn1^iii^	115.8 (2)	C22—C17—S1	118.9 (2)
C1—N1—C5	118.8 (3)	C18—C17—S1	119.8 (2)
C1—N1—Mn1	122.0 (2)	C19—C18—C17	118.9 (3)
C5—N1—Mn1	119.1 (2)	C19—C18—H18A	120.5
C6—N2—C10	118.9 (3)	C17—C18—H18A	120.5
C6—N2—Mn1	116.7 (2)	C18—C19—C20	120.7 (3)
C10—N2—Mn1	124.2 (2)	C18—C19—H19A	119.6
N1—C1—C2	122.7 (3)	C20—C19—H19A	119.6
N1—C1—H1A	118.7	C21—C20—C19	119.4 (3)
C2—C1—H1A	118.7	C21—C20—C24	120.0 (3)
C3—C2—C1	117.9 (4)	C19—C20—C24	120.5 (3)
C3—C2—H2A	121.0	C22—C21—C20	120.5 (3)
C1—C2—H2A	121.0	C22—C21—H21A	119.8
C4—C3—C2	120.0 (3)	C20—C21—H21A	119.8
C4—C3—H3A	120.0	C21—C22—C17	119.6 (3)
C2—C3—H3A	120.0	C21—C22—H22A	120.2
C3—C4—C5	119.8 (3)	C17—C22—H22A	120.2
C3—C4—H4A	120.1	O2—C23—O1	122.2 (3)
C5—C4—H4A	120.1	O2—C23—C11	119.2 (3)
N1—C5—C4	120.8 (3)	O1—C23—C11	118.3 (3)
N1—C5—C6	116.1 (3)	O2—C23—Mn1	68.55 (18)
C4—C5—C6	123.1 (3)	O1—C23—Mn1	55.49 (16)
N2—C6—C7	121.4 (3)	C11—C23—Mn1	159.3 (2)
N2—C6—C5	116.1 (3)	O4—C24—O3	123.6 (3)
C7—C6—C5	122.5 (3)	O4—C24—C20	117.7 (3)
C8—C7—C6	118.2 (4)	O3—C24—C20	118.7 (3)
			
O3^i^—Mn1—O1—C23	−157.81 (19)	C12—C13—C14—C15	0.9 (4)
O4^ii^—Mn1—O1—C23	91.7 (2)	C12—C13—C14—S1	175.4 (2)
N1—Mn1—O1—C23	−26.3 (3)	O6—S1—C14—C15	−169.5 (2)
N2—Mn1—O1—C23	−75.0 (2)	O5—S1—C14—C15	60.0 (3)
O2—Mn1—O1—C23	8.57 (18)	C17—S1—C14—C15	−54.6 (3)
O3^i^—Mn1—O2—C23	29.0 (3)	O6—S1—C14—C13	15.9 (3)
O4^ii^—Mn1—O2—C23	−116.62 (19)	O5—S1—C14—C13	−114.6 (2)
O1—Mn1—O2—C23	−8.64 (18)	C17—S1—C14—C13	130.8 (2)
N1—Mn1—O2—C23	151.4 (2)	C13—C14—C15—C16	−1.0 (5)
N2—Mn1—O2—C23	80.1 (2)	S1—C14—C15—C16	−175.6 (2)
O3^i^—Mn1—N1—C1	−101.3 (2)	C14—C15—C16—C11	0.1 (5)
O4^ii^—Mn1—N1—C1	3.6 (2)	C12—C11—C16—C15	0.9 (5)
O1—Mn1—N1—C1	126.0 (2)	C23—C11—C16—C15	−173.6 (3)
N2—Mn1—N1—C1	177.9 (3)	O6—S1—C17—C22	51.6 (3)
O2—Mn1—N1—C1	97.3 (2)	O5—S1—C17—C22	−178.8 (2)
C23—Mn1—N1—C1	112.2 (2)	C14—S1—C17—C22	−64.2 (3)
O3^i^—Mn1—N1—C5	79.8 (2)	O6—S1—C17—C18	−120.3 (3)
O4^ii^—Mn1—N1—C5	−175.4 (2)	O5—S1—C17—C18	9.4 (3)
O1—Mn1—N1—C5	−53.0 (3)	C14—S1—C17—C18	124.0 (2)
N2—Mn1—N1—C5	−1.1 (2)	C22—C17—C18—C19	−1.5 (5)
O2—Mn1—N1—C5	−81.6 (2)	S1—C17—C18—C19	170.2 (2)
C23—Mn1—N1—C5	−66.8 (2)	C17—C18—C19—C20	−1.2 (4)
O3^i^—Mn1—N2—C6	−99.5 (2)	C18—C19—C20—C21	2.6 (5)
O4^ii^—Mn1—N2—C6	22.3 (5)	C18—C19—C20—C24	−175.7 (3)
O1—Mn1—N2—C6	154.7 (2)	C19—C20—C21—C22	−1.3 (5)
N1—Mn1—N2—C6	2.9 (2)	C24—C20—C21—C22	177.0 (3)
O2—Mn1—N2—C6	97.4 (2)	C20—C21—C22—C17	−1.3 (5)
C23—Mn1—N2—C6	126.2 (2)	C18—C17—C22—C21	2.7 (5)
O3^i^—Mn1—N2—C10	75.5 (3)	S1—C17—C22—C21	−169.0 (2)
O4^ii^—Mn1—N2—C10	−162.7 (3)	Mn1—O2—C23—O1	14.7 (3)
O1—Mn1—N2—C10	−30.3 (3)	Mn1—O2—C23—C11	−158.8 (3)
N1—Mn1—N2—C10	177.9 (3)	Mn1—O1—C23—O2	−16.7 (3)
O2—Mn1—N2—C10	−87.5 (3)	Mn1—O1—C23—C11	156.9 (2)
C23—Mn1—N2—C10	−58.7 (3)	C16—C11—C23—O2	158.7 (3)
C5—N1—C1—C2	0.6 (5)	C12—C11—C23—O2	−15.8 (4)
Mn1—N1—C1—C2	−178.4 (3)	C16—C11—C23—O1	−15.1 (4)
N1—C1—C2—C3	−0.7 (5)	C12—C11—C23—O1	170.5 (3)
C1—C2—C3—C4	0.8 (6)	C16—C11—C23—Mn1	51.5 (7)
C2—C3—C4—C5	−0.8 (6)	C12—C11—C23—Mn1	−122.9 (6)
C1—N1—C5—C4	−0.5 (5)	O3^i^—Mn1—C23—O2	−166.12 (17)
Mn1—N1—C5—C4	178.5 (3)	O4^ii^—Mn1—C23—O2	67.9 (2)
C1—N1—C5—C6	−179.7 (3)	O1—Mn1—C23—O2	164.9 (3)
Mn1—N1—C5—C6	−0.7 (4)	N1—Mn1—C23—O2	−32.3 (2)
C3—C4—C5—N1	0.6 (6)	N2—Mn1—C23—O2	−94.7 (2)
C3—C4—C5—C6	179.7 (4)	O3^i^—Mn1—C23—O1	29.0 (2)
C10—N2—C6—C7	0.4 (5)	O4^ii^—Mn1—C23—O1	−96.9 (2)
Mn1—N2—C6—C7	175.7 (3)	N1—Mn1—C23—O1	162.83 (18)
C10—N2—C6—C5	−179.5 (3)	N2—Mn1—C23—O1	100.5 (2)
Mn1—N2—C6—C5	−4.2 (4)	O2—Mn1—C23—O1	−164.9 (3)
N1—C5—C6—N2	3.3 (4)	O3^i^—Mn1—C23—C11	−49.8 (6)
C4—C5—C6—N2	−175.9 (3)	O4^ii^—Mn1—C23—C11	−175.7 (6)
N1—C5—C6—C7	−176.6 (3)	O1—Mn1—C23—C11	−78.8 (6)
C4—C5—C6—C7	4.3 (5)	N1—Mn1—C23—C11	84.1 (6)
N2—C6—C7—C8	−0.2 (6)	N2—Mn1—C23—C11	21.7 (6)
C5—C6—C7—C8	179.7 (4)	O2—Mn1—C23—C11	116.4 (7)
C6—C7—C8—C9	0.3 (7)	Mn1^iii^—O4—C24—O3	13.8 (4)
C7—C8—C9—C10	−0.7 (7)	Mn1^iii^—O4—C24—C20	−164.7 (2)
C6—N2—C10—C9	−0.7 (5)	Mn1^i^—O3—C24—O4	82.8 (4)
Mn1—N2—C10—C9	−175.7 (3)	Mn1^i^—O3—C24—C20	−98.7 (3)
C8—C9—C10—N2	0.9 (6)	C21—C20—C24—O4	−23.5 (4)
C16—C11—C12—C13	−1.0 (4)	C19—C20—C24—O4	154.9 (3)
C23—C11—C12—C13	173.4 (3)	C21—C20—C24—O3	158.0 (3)
C11—C12—C13—C14	0.1 (4)	C19—C20—C24—O3	−23.7 (4)

Symmetry codes: (i) −*x*+2, −*y*, −*z*+1; (ii) *x*−1, *y*, *z*−1; (iii) *x*+1, *y*, *z*+1.

### Hydrogen-bond geometry (Å, °)

**Table:**

*D*—H···*A*	*D*—H	H···*A*	*D*···*A*	*D*—H···*A*
C1—H1A···O5^iv^	0.93	2.44	3.123 (4)	131
C16—H16A···O6^v^	0.93	2.59	3.424 (4)	149
C19—H19A···O2^vi^	0.93	2.54	3.210 (4)	129
C21—H21A···O5^vii^	0.93	2.51	3.337 (4)	148

Symmetry codes: (iv) *x*−1, −*y*+1/2, *z*−1/2; (v) *x*, −*y*+1/2, *z*−1/2; (vi) *x*+1, −*y*+1/2, *z*+1/2; (vii) *x*, −*y*+1/2, *z*+1/2.
